# Remote myocardial zone characteristics in type 2 diabetes patients with prior myocardial infarction and comparisons with diabetes patients with no prior infarction

**DOI:** 10.1177/20420188241263488

**Published:** 2024-07-24

**Authors:** Sharmaine Thirunavukarasu, Mehak Asad, Sindhoora Kotha, Henry Procter, Marilena Giannoudi, Hui Xue, Peter Kellman, John P. Greenwood, Eylem Levelt

**Affiliations:** Multidisciplinary Cardiovascular Research Centre and Biomedical Imaging Science Department, Leeds Institute of Cardiovascular and Metabolic Medicine, University of Leeds, Leeds, UK; Multidisciplinary Cardiovascular Research Centre and Biomedical Imaging Science Department, Leeds Institute of Cardiovascular and Metabolic Medicine, University of Leeds, Leeds, UK; Multidisciplinary Cardiovascular Research Centre and Biomedical Imaging Science Department, Leeds Institute of Cardiovascular and Metabolic Medicine, University of Leeds, Leeds, UK; Multidisciplinary Cardiovascular Research Centre and Biomedical Imaging Science Department, Leeds Institute of Cardiovascular and Metabolic Medicine, University of Leeds, Leeds, UK; DHHS, National Heart, Lung, and Blood Institute, National Institutes of Health, Bethesda, MD, USA; Multidisciplinary Cardiovascular Research Centre and Biomedical Imaging Science Department, Leeds Institute of Cardiovascular and Metabolic Medicine, University of Leeds, Leeds, UK; DHHS, National Heart, Lung, and Blood Institute, National Institutes of Health, Bethesda, MD, USA; DHHS, National Heart, Lung, and Blood Institute, National Institutes of Health, Bethesda, MD, USA; DHHS, National Heart, Lung, and Blood Institute, National Institutes of Health, Bethesda, MD, USA; Multidisciplinary Cardiovascular Research Centre and Biomedical Imaging Science Department, Leeds Institute of Cardiovascular and Metabolic Medicine, University of Leeds, Leeds, UK; Baker Heart and Diabetes Institute, Monash University, Melbourne, VIC, Australia; Multidisciplinary Cardiovascular Research Centre and Biomedical Imaging Science Department, Leeds Institute of Cardiovascular and Metabolic Medicine, University of Leeds, Leeds, LS29JT, UK

**Keywords:** cardiovascular magnetic resonance (CMR) imaging, diabetes, heart failure (HF), myocardial infarction

Heart failure (HF) is the leading cause of mortality in patients with type 2 diabetes (T2D). There is a strong synergy between coronary artery disease (CAD) and diabetes, with a 2- to 4-fold higher risk of myocardial infarction (MI) in T2D patients compared to the general population.^1^ The presence of ischaemic scar has been shown to be the strongest predictor of outcome in T2D patients with prior MI (*prior*MI-DM).^1^ Moreover, ventricular dysfunction has been identified in T2D patients in the absence of CAD or prior MI. While strict risk-factor control in T2D patients successfully modifies the risk of MI, the excess risk of HF persists.^2^

Cardiovascular magnetic resonance (CMR) imaging plays a pivotal role in the assessment of the MI sequelae allowing for quantification of the infarct size, evaluation of post-MI left ventricular (LV) remodelling and detection of other complications of MI.^3^

Utilizing CMR, we assessed the remote myocardial zone contractile function (circumferential systolic strain; *remote*CSS), rest and stress myocardial blood flow (*remote*MBF) and extra-cellular volume fraction (*remote*ECV) in *prior*MI-DM patients, and compared these remote zone assessments to the global circumferential systolic strain and rest and stress MBF and ECV in T2D patients with no prior MI (*no*MI-DM) and healthy volunteers (HV). Remote zone was defined as myocardial tissues unaffected by late gadolinium enhancement (LGE) and located opposite to the infarct zone on the AHA 16 segment model. We further assessed the relationship of the infarct size and the glycemic control in diabetes with the cardiac functional remodelling by assessing bivariate correlations of these parameters with markers of global cardiac contractile function [global longitudinal strain (GLS) and left ventricular ejection fraction (LVEF)].

Participants with similar age, sex and body mass index distribution (42 *prior*MI-DM, 47 *no*MI-DM and 22 HVs) were recruited. Subjects underwent CMR (3T) for cine, rest and adenosine stress pixel-wise perfusion mapping and LGE imaging. None of the participants had a clinical diagnosis of HF. All *prior*MI-DM patients had evidence of MI on LGE and had received full percutaneous revascularization treatment >12 months prior to study recruitment. Thirteen had a diagnosis of non-ST-elevation MI, 22 ST-elevation MI and 7 silent MI with left anterior descending territory MI present in 18 patients, right coronary artery territory MI in 11 patients and circumflex territory MI in 13 patients. Seven percent of the patients had more than eight segments involved, 24% four to eight segments involved, and 69% less than four segments infarcted. The mean total segment involvement was 19%.

Demographics, biochemical and CMR data (means and 95% confidence intervals) are shown in Table 1.

**Table 1.** Demographics, biochemical and CMR characteristics.

Variables	Controls (*n* = 22)	*prior*MI-DM (*n* = 42)	*no*MI-DM (*n* = 47)	ANOVA
Age (years)	66 (63–70)	67 (62–69)	67 (57–72)	0.8
Male (*n*, %)	17 (77)	30 (72)	34 (72)	0.5
Smoking (*n*, %)	2 (10)	19 (45)	19 (40)	0.2
Body mass index (kg/m^2^)	26 (25–28)	28 (27–30)	28 (27–30)	0.08
Glucose (mmol/l)	5.2 (4.8–5.2) Ψα	8.7 (7.4–10) α	9.1 (8–10) Ψ	<0.001
Glycated haemoglobin (mmol/mol)	38 (36–39) Ψα	60 (54–62) α	64 (59–70) Ψ	<0.001
Systolic BP (mmHg)	127 (123–132)	124 (118–131)	130 (125–135)	0.5
Diastolic BP (mmHg)	78 (74–77)	74 (71–77)	75 (73–77)	0.3
Cardiac structural and functional comparisons				
LV end-diastolic volume index (ml/m^2^)	80 (74–86)	78 (72–83)	78 (72–83)	0.7
LV end-systolic volume index (ml/m^2^)	30 (27–33)	34 (30–39)	36 (31–41)	0.2
LV stroke volume (ml)	98 (88–107) Ψ	85 (78–93)	82 (77–86) Ψ	0.01
LV ejection fraction (%)	63 (61–65) Ψα	56 (53–59) α	55 (52–58) Ψ	0.002
LV mass (g)	105 (100–110)	107 (98–116)	102 (94–110)	0.7
LV mass/LV end-diastolic volume (mg/ml)	0.7 (0.6–0.8)	0.7 (0.6–0.8)	0.7 (0.6–1)	0.9
Remote myocardium circumferential strain (%)	−21 (−20 to 22) Ψ	−20 (−19 to 21)	−19 (−18 to 20) Ψ	0.01
Global longitudinal strain (%)	−15 (−14 to 16) Ψα	−12 (−11 to 13) α	−11 (−10 to 13) Ψ	<0.001
LGE (g)	–	9.7	–	**–**
Rest and stress perfusion and RPP				
Stress RPP (bpm * mmHg)	11,942 (9754–14,129)	8455 (6973–9938)	11,198 (10,174–12,222)	0.7
Rest RPP (bpm * mmHg)	8339 (7381–9297)	6531 (5395–8203)	8764 (8203–9325)	0.7
Global stress MBF (ml/g/min)	1.9 (1.7–2)	1.7 (1.4–1.8)	1.8 (1.7–1.9)	0.7
Global rest MBF (ml/g/min)	0.7 (0.64–0.74)	0.6 (0.6–0.7)	0.7 (0.6–0.8)	0.1
Myocardial perfusion reserve	3.3 (2.5–2.9)	2.4 (2.1–2.7)	2.8 (2.4–3)	0.3

Values are mean [LL of 95% confidence interval − UL of 95% confidence interval].

α indicates *p* < 0.05 between controls and *prior*MI-DM. Ψ indicates *p* < 0.05 between controls and *no*MI-DM. Ω indicates *p* < 0.05 between *priorM*I-DM and *noMI*-DM.

ANOVA, analysis of variance; bpm, beats per minute; BP, blood pressure; CMR, cardiovascular magnetic resonance; g, gram; g/m^2^, gram per square metre of body surface area; LGE, late gadolinium enhancement; LL, lower limit; LV, left ventricular; MBF, myocardial blood flow; min, minute; ml, millilitre; ml/m^2^, millilitres per square metre of body surface area; mmHg, millimetres of mercury; RPP, rate pressure product; UL, upper limit.

There were no significant differences in BP, diabetes duration, treatment or HbA1c between the two diabetes groups. Participants from all three groups demonstrated a similar increase in rate pressure product during adenosine stress. When compared to controls, both diabetes groups showed similar reductions in LVEF (Table 1). Biventricular volumes, rest MBF and myocardial perfusion reserve were similar across the groups. Stress MBF was significantly decreased in the remote myocardium zone in *prior*MI-DM group while global stress MBF was not significantly decreased in either diabetes groups [remote zone *prior*MI-DM 1.7 (1.4–1.8) *versus* global *no*MI-DM 1.8 (1.7–1.9) *versus* global HV 1.9 (1.7–2) ml/g/min, *p* = 0.1]. The *remote*ECV and the *global*ECV were similar in *prior*MI-DM group and these were significantly increased compared to noMI-DM and HV groups [remote zone *prior*MI-DM 26 (25–28) *versus* global *no*MI-DM 23 (22–23) *versus* global HV 24 (23–25)%, *p* = 0.002].

The *remote*ECV correlated with infarct size (*r* = 0.66, *p* = 0.001). In the *prior*MI-DM group, only the infarct size correlated with the GLS (*r* = −0.4, *p* = 0.005), not the HbA1c. However, there was an inverse correlation between the HbA1c and the GLS in *no*MI-DM group (*r* = −0.3, *p* = 0.03).

In conclusion, diabetes patients exhibit significant alterations in LVEF and GLS even in the absence of prior MI. *Prior*MI-DM patients show significant alterations in remote myocardial zone with lower circumferential strain and stress MBF, and higher ECV than *no*MI-DM group, even in patients who have received full revascularization. In *prior*MI-DM patients, the infarct size correlated significantly with global contractile function; however, HbA1c correlated with GLS only in *no*MI-DM patients. Hence, poor glycemic control may more strongly underpin myocardial function in diabetes patients without prior MI, whereas other factors appear to be more important in the context of prior MI and primary prevention of ischaemic heart disease risk remains an important goal in diabetes.

## Declarations

**Ethics approval and consent to participate:** This single-centre prospective, longitudinal, cohort study complied with the Declaration of Helsinki. It was approved by the National Research Ethics Committee (REC Ref: 18/YH/0168) and informed written consent was obtained from each participant.

**Consent for publication:** No patient images were used as a figure for publication.

**Availability of data and materials:** The data will be shared on reasonable request to the corresponding author.

## References

[1] Kwong Raymond Y Sattar H Wu H , et al. Incidence and prognostic implication of unrecognized myocardial scar characterized by cardiac magnetic resonance in diabetic patients without clinical evidence of myocardial infarction. Circulation 2008; 118: 1011–1020. 18725488 10.1161/CIRCULATIONAHA.107.727826 PMC2743310

[2] Rawshani A Rawshani A Franzén S , et al. Risk factors, mortality, and cardiovascular outcomes in patients with type 2 diabetes. N Engl J Med 2018; 379: 633–644. 30110583 10.1056/NEJMoa1800256

[3] Podlesnikar T Pizarro G Fernández-Jiménez R , et al. Left ventricular functional recovery of infarcted and remote myocardium after ST-segment elevation myocardial infarction (METOCARD-CNIC randomized clinical trial substudy). J Cardiovasc Magn Reson 2020; 22: 44. 32522198 10.1186/s12968-020-00638-8 PMC7288440

